# Analysis of Surface Texture Distribution Characteristics of Concrete Substrate and Modeling of Coating Adhesion Strength

**DOI:** 10.3390/ma18235412

**Published:** 2025-12-01

**Authors:** Tao Fan, Peng Xu, Huaxin Chen, Teng Yuan, Anhua Xu, Cheng Chen, Yongchang Wu

**Affiliations:** 1 School of Materials Science and Engineering, Chang’an University, Xi’an 710061, China; fantao1102@chd.edu.cn (T.F.);; 2 Xi’an Highway Research Institute Co., Ltd., Xi’an 710065, China; 3 Xi’an Huaze Highway Material Co., Ltd., Xi’an 710065, China; 4 Xinjiang Transportation Planning Survey and Design Institute Co., Ltd., Urumqi 830063, China; 5 Xinjiang Key Laboratory for Safety and Health of Transportation Infrastructure in Alpine and High-Altitude Mountainous Areas, Urumqi 830063, China; 6 School of Hydraulic and Civil Engineering, Qinghai Vocational and Technical University, Xining 810003, China; 7 School of Highway, Chang’an University, Xi’an 710064, China

**Keywords:** concrete surface texture, concrete coating adhesion performance, three-dimensional roughness, correlation coefficient

## Abstract

This study systematically investigates the influence of concrete substrate surface texture characteristics on the adhesion strength of waterborne epoxy coatings. By employing surface treatment techniques such as brush scouring and grinding, the surface roughness, pore structure, and three-dimensional morphology of concrete substrates were quantitatively analyzed using laser scanning and parameter modeling. The correlation between texture parameters (e.g., three-dimensional arithmetic mean roughness S_a_ and proportion of the hole area) and coating adhesion strength under varying curing temperatures (−18 °C, 20 °C, 40 °C, and 60 °C) was evaluated through pull-off tests and Pearson Correlation Analysis. Results indicate that the absolute proportion of hole area (A_a_) (r ≈ −0.93) and S_a_ (r ≈ −0.81) are key factors affecting adhesion. Surface treatments, including 5 h scouring and 40 min grinding, enhanced pull-off strength by 40–60% by optimizing mechanical interlocking. A nonlinear regression model was established to predict adhesion strength, suggesting an operational S_a_ range of approximately 0.35–0.40 mm for the current coating system at 20 °C. Temperature significantly modulated the adhesion mechanism; low-temperature curing exacerbated pore defects, while high-temperature conditions intensified thermal stress. Practical guidelines to improve permeability include optimizing the surface roughness through grinding, strictly controlling the absolute proportion of holes, and using preheated or low-viscosity resin in a low-temperature environment.

## 1. Introduction

Concrete surface coatings are widely applied to enhance structural durability and provide protection against environmental degradation [1,2]. The penetration behavior of coatings is primarily governed by their macroscopic properties, such as viscosity and flow characteristics [3,4]. However, the adhesion performance of coatings is critically influenced by substrate surface characteristics [5], including roughness [6], porosity, and pore distribution [7]. Traditional roughness studies mainly rely on two-dimensional roughness parameters (such as the arithmetic mean deviation of the profile Ra) to characterize concrete surface features [8]. However, such parameters cannot comprehensively reflect the actual three-dimensional topography of the substrate and the coating penetration potential. Recent advances in digital image processing and fractal theory [9] have enabled more refined and multi-dimensional characterization of surface texture. Although existing studies [10,11,12,13] have focused on two-dimensional roughness parameters (e.g., R_a_), the three-dimensional texture features and their synergistic effects with environmental factors like temperature remain underexplored [14].

Traditional surface preparation methods, such as scouring and grinding, alter the substrate’s micro- and macro-texture, thereby affecting the mechanical interlocking between the coating and concrete [10]. However, the quantitative relationship between these texture modifications and adhesion strength lacks systematic characterization. Furthermore, temperature variations during curing can significantly alter resin fluidity and interfacial stress distribution, potentially compromising adhesion [15,16,17,18,19]. Addressing these gaps is essential for optimizing coating processes in diverse temperature conditions.

This study aims to characterize the three-dimensional texture parameters of concrete substrates post surface treatments (scouring and grinding) using laser scanning and statistical analysis, investigate the correlation between texture parameters (e.g., three-dimensional arithmetic mean roughness S_a_ and proportion of the hole area) and epoxy coating adhesion strength under varying curing temperatures, and develop predictive models to guide surface pretreatment and temperature-adaptive coating applications.

The experimental framework includes surface morphology analysis, pull-off strength testing, and nonlinear regression modeling. The findings provide practical insights into texture parameter optimization and temperature control, advancing the engineering application of durable concrete coatings.

## 2. Experimental Section

### 2.1. Materials

The substrate specimens were prepared using C40 concrete. To facilitate coating application tests, the original concrete blocks (600 mm × 150 mm × 150 mm) were sectioned into smaller specimens (100 mm × 150 mm × 150 mm) using a diamond cutting machine.

The concrete mix proportion was formulated using the following components per cubic meter: cement: 360 kg; sand: 675 kg; coarse aggregate: 1150 kg/m^3^; and water: 215 kg/m^3^.

The water-to-cement ratio (W/C) was maintained at 0.6, with a sand-to-aggregate ratio of 35–40%. After 28 days of standard curing, the specimens were cleaned to remove surface dust, embedded debris, and loose particles. The demolded surfaces of the original specimens served as test surfaces, with four test surfaces available per specimen. To mitigate the influence of cutting-induced microstructural variations on absorption properties, the cut surfaces were sealed with an epoxy sealer at a coating rate of 180–200 g/m^2^.

### 2.2. Coatings and Coating Methods

The coating material consisted of a custom-formulated waterborne epoxy varnish. The waterborne epoxy resin emulsion (industrial-grade LJ910) and corresponding curing agent (industrial-grade GEM03) were procured from Shanghai Lüjia Waterborne Coatings Co., Ltd. (Shanghai, China). Deionized water was produced in-house using a laboratory deionization system.

The coating formulation process consisted of the following steps: Mixing protocol: Precise ratios of waterborne epoxy resin, curing agent, and deionized water were blended using mechanical agitation (500 rpm for 2 min); Aging phase: The mixture was aged for 3 min under ambient conditions (23 ± 1 °C, 50 ± 5% RH); and Coating application: Uniform films were deposited using a wire-wound applicator (100 μm clearance) with a controlled spread rate of 120–150 g/m^2^. Ensure that the dry film thickness of the coating is approximately 80 μm.

### 2.3. Test Methods

#### 2.3.1. Grinding Test

To simulate severely corroded/carbonized surfaces, manual grinding was performed using a Delixi Electric (Hangzhou, China) DLX-SIM-HST-100C angle grinder.

The abrasive processing employed a 100 mm diameter steel wire cup brush operating at 11,000 rpm no-load speed with 710 W power input. The standardized grinding procedure comprised: (1) specimen fixation using rubber-backed worktables (Shore A 60 hardness); (2) linear reciprocating motion with 50 mm path overlap; and (3) contact pressure maintained at 20 N.

#### 2.3.2. Roughness Characterization

(1) Interfacial Roughness and Water Absorption

Non-testing surfaces were sealed with waterborne epoxy primer (dry film thickness: 50 ± 5 μm) and cured at 23 ± 2 °C for 72 h. Water immersion testing commenced after verifying coating integrity and leak detection.

Water absorption rate (WAR) and water absorption per unit area (WA) were calculated as:

(1) $$\mathrm{WAR}(\%)=\frac{M_{1}-M_{0}}{M_{0}}\times 100\%$$

(2) $$\mathrm{WA}=\frac{M_{1}-M_{0}}{A}$$

where *M*_0_—mass of concrete specimens before soaking (g); *M*_1_—mass of concrete specimens after soaking (g); and *A*—test piece soaking area (mm^2^).

(2) Silica Powder Roughness Index (MTD)

The Mean Texture Depth (MTD) was quantified using silicon powder particle heap state (ASTM E965) using the following formula:

(3) $$MTD=V_{V}/A$$

where *MTD*—average texture depth (mm); *V_V_*—volume of silica fume; and *A*—coverage area.

(3) Stylus Roughness Profiling

Stylus Roughness index (SRI) was measured using a Mitutoyo Surftest SJ-410 profilometer (Juechang Instrument Measurement (Shanghai) Co., Ltd., Shanghai, China) according to the ISO 8503 standard. The tip radius was 5 µm, measured using a non-sliding method. The test results are shown in Table 1.

(4) $$SRI=\frac{1}{n}\sum_{i=1}^{n}D_{i}$$

In the formula: *SRI*—Stylus Roughness index (mm); *n*—the total number of measurement points within the test area grid; and *D_i_*—depth measurement value of the i-th measurement point (mm).

#### 2.3.3. 3D Surface Topography Modeling

**Figure 1:**
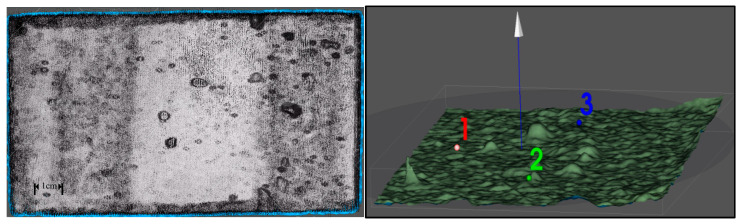
Laser scanning process and normal vector assignment of concrete surface.

Surface topography was mapped non-destructively using a Seal Lite-3D laser scanner (Hexagon Metrology, Shenzhen, China) configured with a 0.02 mm resolution, 100,000 pts/s acquisition rate, and 21.17 μm pixel pitch. Before scanning, ensure automatic focusing during the scanning process through trial scanning, and calibrate and verify the total number of scanning frames. All scans were conducted in a controlled laboratory environment (temperature 20 ± 2 °C and relative humidity 50 ± 5%) to minimize environmental interference during scanning. The scanning position should be maintained at approximately 15 to 20 cm in front of the specimen. During scanning, the continuous scanning duration should not be less than 2 min and the total number of frames should not be less than 1000. A single test surface should be scanned from different angles for more than three overlapping scans. The raw point cloud data acquired by the laser scanner were processed using JMStudio software (Version 2.6.3.0146) with a primary filtering algorithm (neighborhood points: 50; outlier standard deviation threshold: 3.0) to remove noise, followed by surface reconstruction in Geomagic Studio 2021 incorporating mesh optimization (50 μm target triangle edge length) and boundary trimming (0.1 mm tolerance), and surface leveling and reference plane selection, culminating in OBJ-format export with vertex normal vectors. The scanning process is shown in Figure 1.

OBJ file vertices were parsed to extract Cartesian coordinates (x, y and z) with the *z* axis representing depth [20,21,22]. As shown in Figure 1, in the grid plane, select points 1 to 3 as reference points to determine the reference plane and the normal vector of the reference plane. Surface normals were calculated using a weighted least squares algorithm (neighborhood radius: 0.5 mm).

### 2.4. Calculation of 3D Surface Texture Parameters

Calculation of the three-dimensional arithmetic average roughness (S_a_, mm): S_a_ is used to represent the average value of the absolute deviation of all points on the substrate from the mean plane, similar to the two-dimensional arithmetic average roughness (R_a_), but more comprehensively reflecting the overall roughness [23]. The calculation formula is shown in (5).

(5) $$S_{a}=\frac{1}{n}\sum_{i=1}^{n}\left|z_{i}\right|$$

Calculation of the three-dimensional maximum depth (S_z_, mm): S_z_ is used to represent the vertical distance between the highest peak and the deepest valley in the substrate area. It is a positive-valued parameter by definition (S_z_ ≥ 0), quantifying the extreme depth of surface valleys or pores. This parameter should not be confused with the standardized height parameter S_z_ in ISO 25178.

Calculation of the three-dimensional average depth (S_av_, mm): S_av_ is used to calculate the arithmetic mean depth of all points on the substrate surface.

Calculation of the absolute proportion of hole area (A_a_) and relative proportion of hole area (A_r_): the ratio of the number of coordinates with a depth value greater than 0.3 mm (z > 0.3 mm) to the total number of coordinates, and the ratio of the number of coordinates with a depth value greater than the average depth value of 0.3 mm (z > $\bar{z}$ + 0.3 mm) to the total number of coordinates, respectively. Since approximately 98% of the undulations of planar surfaces are within 0.3 mm, to distinguish between surface roughness and pores, a critical threshold of 0.3 mm was selected based on preliminary experiments for evaluating the pore condition.

Calculation of the root mean square roughness (S_q_): S_q_ is used to represent the root mean square value of the height of each point, reflecting the distribution characteristics of the peak–valley changes on the surface, and a larger S_q_ value indicates that the surface undulation is more intense [24]. The calculation formula is shown in (6).

(6) $$S_{q}=\sqrt{\frac{1}{n}\sum_{i=1}^{n}{\left(z_{i}-\bar{z}\right)}^{2}}$$

In the formula: *z_i_*—the depth value of the i-th sampling point; *n*—total number of points; and $\bar{z}$—three-dimensional average depth.

Calculation of skewness (S_sk_): S_sk_ is used to represent the symmetry of the height distribution of each point. When S_sk_ > 0, the surface is dominated by the peak structure generated by the cutting face; when S_sk_ < 0, the surface is dominated by the valley structure generated by thermal action; and S_sk_ = 0 indicates a symmetrical distribution [25]. The calculation formula is shown in (7).

(7) $$S_{sk}=\frac{1}{n{S_{q}}^{3}}\sum_{i=1}^{n}{\left(z_{i}-\bar{z}\right)}^{3}$$

Calculation of sharpness (S_ku_): S_ku_ is used to represent the sharpness of the distribution of height at each point. S_ku_ = 3 represents a normal distribution, S_ku_ > 3 indicates a distribution curve that is sharper (such as a polished surface), and S_ku_ < 3 indicates a flatter distribution (such as a sandblasted surface) [26]. The calculation formula is shown in (8).

(8) $$S_{ku}=\frac{1}{n{S_{q}}^{4}}\sum_{i=1}^{n}{\left(z_{i}-\bar{z}\right)}^{4}$$

Calculation of Pearson correlation coefficient (*r*): *r* is used to analyze the correlation between two variables through the correlation coefficient, and the calculation formula is shown in (9). All reported correlations are Pearson correlation coefficients. The sample size at each temperature is 15, with a 95% confidence interval. The relevant results presented here are used only for the preliminary selection of regression modeling parameters; therefore, no false discovery rate (FDR) correction was performed.

(9) $$r=\frac{\sum_{i=1}^{n}\left(X_{i}-\bar{X})(Y_{i}-\bar{Y}\right)}{\sqrt{\sum_{i=1}^{n}{\left(X_{i}-\bar{X}\right)}^{2}•\sqrt{\sum_{i=1}^{n}{\left(Y_{i}-\bar{Y}\right)}^{2}}}})$$

In the formula: *X_i_*, *Y_i_*—measured values of the two variables; $\bar{X}$,$\bar{Y}$—the mean of the variables; and *n*—Total sample size.

### 2.5. Pull-Off Strength of Substrate and Coating

**Figure 2:**
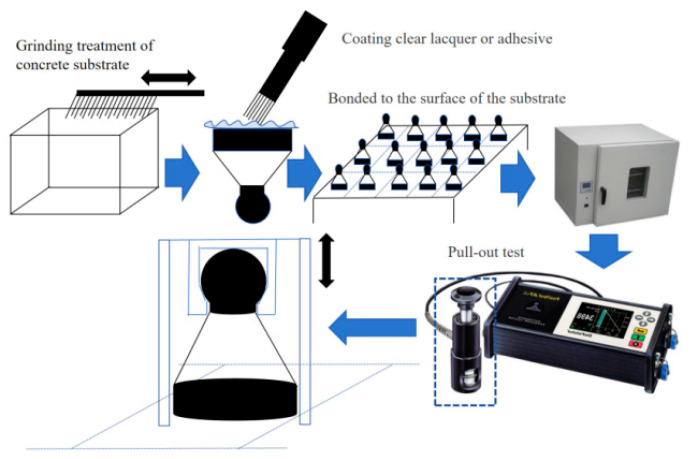
Post-test-A pull strength meter test.

The pull-off strength was tested using a post-test-A pull-off strength instrument by bonding a rod ingot to a dolly (ASTM D4541) (Figure 2). The bonding test was carried out with a bonding strength tester.

Following the modeling process, the concrete specimens were placed in environmental chambers set at varying temperatures and subjected to a curing period of at least 4 h to guarantee consistency with the ambient temperature. They were then removed, and the coating was applied within 5 min. The coated specimens (with uncured surfaces) were subsequently returned to the environmental chambers to undergo surface drying and thorough drying/curing. After curing for 24 h, the test dollies were bonded to the coating. The specimens continued to cure in the same environmental chambers for an additional 24 h. Upon removal, the pull-off test on the dollies was conducted immediately and completed within 3 min. To ensure the pull-off test accurately measured the adhesion capability between the coating film and the concrete substrate, the area of coating film pulled off by the dolly had to exceed 90%. Partial penetration of the coating film into the porous structure of the concrete was permissible.

The formulation of the water-based epoxy coating used for application was consistent. The viscosity of the mixed coating was approximately 45 s (measured by a No. 4 cup Krebs Stormer viscometer at 25 °C). The coating thickness ranged from 50 to 70 μm, with an application rate of 200 g per square meter.

The coating around the pull-off instrument disk seat was cut off with an a-type cutter, and only the coating near the mandrel was left. The pull-off strength tester relative humidity was (50 ± 5)%. The pulling speed was not more than 0.5 MPa/s.

## 3. Results and Discussion

### 3.1. Cement Coatings Morphology

#### 3.1.1. Morphology and Texture of Concrete Under Grinding

**Figure 3:**
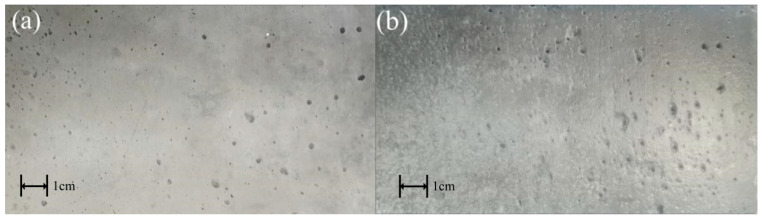
Comparison before and after grinding. (**a**) Unpolished and (**b**) grinding time 20 min.

The macroscopic interfacial morphology of the concrete specimen after the abrasion test is shown in Figure 3:

A comparison of before and after abrasion reveals significant changes in the overall morphology of the concrete substrate. The pattern of changes resembles that observed during flushing, but the abrasion marks are more pronounced. Following abrasion, the pores in the concrete specimen were enlarged, while surface micro-pores progressively expanded during the grinding process. The steel wire left multiple scratches in the abraded area.

After abrasion treatment, the concrete surface exhibited a rougher texture, with originally smooth areas replaced by distinct scratches and uneven textures. These abrasion marks not only increased the surface roughness but also exposed previously sealed micro-pores, creating additional permeation channels. These newly formed channels provide conducive conditions for coating penetration and anchoring, thereby enhancing the mechanical interlocking effect between the coating and the substrate.

#### 3.1.2. Three-Dimensional Morphology of Concrete Substrate Surface

**Figure 4:**
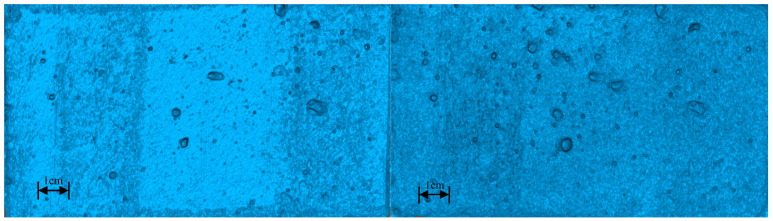
Modeling morphology of the specimen before and after grinding.

Following the abrasion treatment, the surface morphology of the prepared concrete was characterized, and its three-dimensional topographic model was generated through modeling techniques in Figure 4.

The 3D simulation visually demonstrates significant morphological differences between pre-abrasion and post-abrasion substrates. Localized details reveal characteristic changes including pore expansion and increased scratch density, accurately replicating the actual surface conditions.

The 3D topographic model of the abraded surface was sectioned into specimens for pull-off testing. Based on the segmented 3D testing units, distinct morphological features were observed across different testing locations on the same abraded surface. Significant variations exist in surface parameters such as pore area distribution and cavity depth profiles. These spatial heterogeneities necessitate further analysis of how specific morphological parameters influence adhesion performance.

### 3.2. Relationship Between Surface Texture Parameters and Drawing Strength of Concrete Substrate at Different Temperatures

By testing the distribution patterns of texture feature parameters and pull-off strength (POS) at different curing temperatures, the relationship between these parameters and pull-off strength at various temperatures was analyzed. This includes analysis of the Stylus Roughness index (SRI), three-dimensional arithmetic average roughness (S_a_), root mean square roughness (S_q_), three-dimensional maximum depth (S_z_), skewness (S_sk_), sharpness (S_ku_), and absolute proportion of hole area and relative proportion of hole area (A_a_, A_r_).

#### 3.2.1. The Relationship Between SRI, S_av_, and POS at Different Curing Temperatures

**Figure 5:**
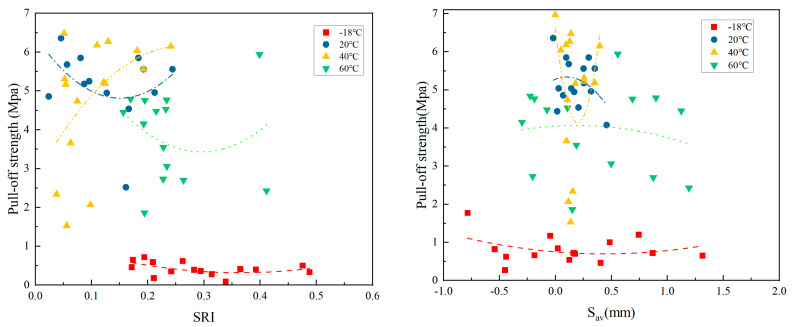
Relationship between roughness index, three-dimensional average depth, and drawing strength of probes at different temperatures.

The relationship between the contact point roughness index and POS at different curing temperatures is shown in Figure 5.

As shown in Figure 5, the SRI of the contact needle at −18 °C ranged from 0.17 to 0.48, with a pull-off strength of 0.09–0.72 MPa; at 20 °C, the SRI ranged from 0.02 to 0.24, with a POS of 2.34–6.36 MPa; at 40 °C, the SRI ranged from 0.04 to 0.24, with a POS of 1.53–6.48 MPa; and at 60 °C, the SRI ranged from 0.16 to 0.41, with a POS of 1.86–5.94 MPa. Compared to the POS being below 1 MPa after curing at −18 °C, the POS distribution after curing at temperatures above 20 °C was mainly between 4 and 6 MPa, and the number of points with a POS between 2 and 4 MPa gradually increased at temperatures above 40 °C.

The Pearson correlation coefficient between the roughness index and drawing strength at different temperatures was calculated, and the results showed that *r* (−18 °C) ≈ −0.82, showing a strong negative correlation; *r* (20 °C) ≈ −0.76, showing a strong negative correlation; *r* (40 °C) ≈ −0.38, showing a weak negative correlation; and *r* (60 °C) ≈ −0.65, showing a moderate negative correlation.

Although the POS at low temperatures decreased slowly with an increase in the roughness index, the overall trend is significant. The POS is 0.46 MPa at an SRI of 0.1716, and the POS is 0.33 MPa at an SRI of 0.4879. During the low-temperature curing process, the resin’s poor fluidity made it difficult to fully fill the peaks and valleys on the rough surface, reducing the effective contact area and leading to stress concentration.

The trend of tensile strength at room temperature is similar to that at −18 °C, but the overall strength is significantly higher, ranging from 4.0 to 5.5 MPa. This indicates that the resin has a higher degree of curing at room temperature, leading to the formation of more bonding groups. As the temperature increases, the fluidity during curing is further improved, although excessive roughness can limit the coating’s permeability. At 40 °C, the correlation between temperature and drawing strength is weak, but there are still some data points of high contact point roughness index, and the strength is still high. This may be because the increase in temperature further optimizes the resin flowability, and the roughness defects are partially compensated. The curing rate of resin and curing agent plays a dominant role in the drawing strength. At 60 °C, its tensile strength is moderately negatively correlated. Compared to the concentrated distribution at normal temperatures, there is an abnormal phenomenon where the adhesion strength remains low despite high roughness. This could be due to uneven thermal contraction leading to localized strengthening. It suggests that high temperatures accelerate curing but may also trigger thermal stress, with rough surfaces and contraction working together to amplify defect effects.

The distribution patterns of the three-dimensional average depth and drawing strength, as well as those of the contact tip roughness index and drawing strength, are largely consistent. The contact tip roughness index and the three-dimensional average depth both indicate the presence of unfilled defects on the substrate surface to some extent, which together reduce adhesion. At temperatures below −18 °C, the POS is below 2 MPa; at temperatures above 20 °C, the POS ranges from 4 to 6 MPa; and at temperatures above 40 °C, the POS points show greater dispersion. By calculating the Pearson correlation coefficients between the POS and the three-dimensional average depth for each temperature group, the results show that *r* (−18 °C) ≈ −0.68, indicating a moderate negative correlation; *r* (20 °C) ≈ −0.45, indicating a weak negative correlation; *r* (40 °C) ≈ 0.12, indicating no significant correlation; and *r* (60 °C) ≈ −0.53, indicating a moderate negative correlation.

At −18 °C, the three-dimensional average depth increases, and the POS decreases. During low-temperature curing, the resin’s fluidity is poor, leading to the formation of void defects in larger surface structures, which reduces the effective contact area. At 20 °C, some data points with higher depths still maintain high strength. At room temperature, the resin’s fluidity improves, but excessive depth can still impede coating penetration. At 40 °C, the point distribution is scattered, with no significant correlation, but the temperature can further optimize the resin’s filling ability. The impact of three-dimensional depth is compensated by fluidity, and the degree of curing uniformity becomes the primary factor affecting drawing strength. At 60 °C, the increase in depth leads to a decrease in strength, and there is a phenomenon where high-depth areas have low adhesion strength. This may be due to resin shrinkage caused by high temperatures, which intensifies thermal stress concentration on larger surfaces, leading to local peeling.

#### 3.2.2. The Relationship Between S_a_, S_q_, and POS at Different Curing Temperatures

**Figure 6:**
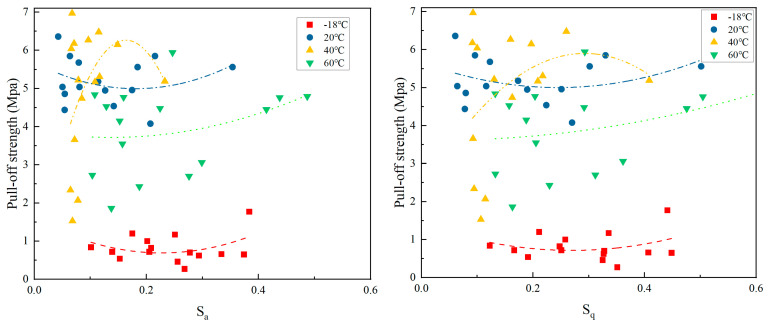
The relationship between the three-dimensional arithmetic mean roughness, root mean square roughness, and drawing strength.

The relationship between the three-dimensional arithmetic mean roughness, root mean square roughness, and drawing strength at different curing temperatures is shown in Figure 6.

As shown in Figure 6, S_a_ (mm) at temperatures of −18 °C, 20 °C, 40 °C, and 60 °C is 0.10~0.38, 0.04~0.35, 0.07~0.23, and 0.10~0.49, respectively. The Pearson correlation coefficients between POS and S_a_ for each temperature group are as follows: *r* (−18 °C) ≈ −0.82, indicating a strong negative correlation; *r* (20 °C) ≈ −0.75, showing a strong negative correlation; *r* (40 °C) ≈ −0.35, indicating a weak negative correlation; and *r* (60 °C) ≈ −0.58, indicating a moderate negative correlation.

When S_a_ increases, the POS decreases significantly. When curing at a low temperature, the resin has poor fluidity, and the peaks and valleys of the rough surface are not fully filled, resulting in the reduction in effective contact area and local stress concentration.

The conditions above 20 °C are similar to those at −18 °C, but the overall intensity is higher. At temperatures above room temperature, the resin’s fluidity improves, but high S_a_ levels still limit coating penetration and weaken mechanical interlocking. At 40 °C, medium temperature, there is even a weak negative correlation, with no significant relationship, and some high S_a_ data points show higher drawing strength. At medium temperature, the resin’s fluidity is optimized, and the impact of surface roughness is partially compensated, with curing uniformity being the dominant factor. There is a medium negative correlation between 60 °C and high temperature. The increase in S_a_ leads to a decrease in strength, but when there is a low S_a_ value, the POS is high, indicating that under high temperature conditions, the curing process will accelerate the shrinkage, and at the same time, the higher S_a_ value surface will further aggravate the concentration of thermal stress, thus causing a local peeling phenomenon.

At both low and high temperatures, S_a_ and SRI are strongly negatively correlated with adhesion strength (*r* ≈ −0.8), indicating that the mechanisms by which different surface roughness parameters (S_a_ and SRI) affect adhesion strength are largely consistent. However, at all temperatures, S_a_ is negatively correlated with drawing strength, but the correlation weakens as temperature increases (−18 °C > 20 °C > 60 °C > 40 °C). At low temperatures, S_a_ is a primary influencing factor, while at medium and high temperatures, other parameters such as fluidity and thermal stress become more significant. Therefore, in low-temperature conditions, it is crucial to prioritize S_a_.

At different curing temperatures, the relationship between S_q_ and POS is similar to that of S_a_ and POS. At −18 °C, there is a strong negative correlation (*r* ≈ −0.85). As S_q_ increases, POS significantly decreases. This suggests that during low-temperature curing, the resin’s poor fluidity results in insufficient filling of the peaks and valleys on a highly rough surface (i.e., a surface with significant undulations), leading to localized stress concentration points and reducing the effective contact area.

At 20 °C, a strong negative correlation (*r* ≈ −0.78) is observed at room temperature, indicating that the resin’s fluidity improves to some extent. However, the high S_q_ value still limits the coating’s permeability and weakens the mechanical interlocking effect. At 40 °C, a weak negative correlation (*r* ≈ −0.32) is observed at medium temperature, with no significant correlation. Some high S_q_ points still exhibit high strength, and the resin’s fluidity is optimized at medium temperature, partially compensating for the impact of S_q_. At 60 °C, a moderate negative correlation (*r* ≈ −0.61) is observed at high temperature, with an increase in S_q_ leading to a decrease in strength and multiple discrete abnormal points. This may be due to accelerated curing shrinkage at high temperatures, which intensifies thermal stress concentration on the high S_q_ surface, causing local peeling.

#### 3.2.3. The Relationship Between S_z_, S_sk_, S_ku_, and POS at Different Curing Temperatures

**Figure 7:**
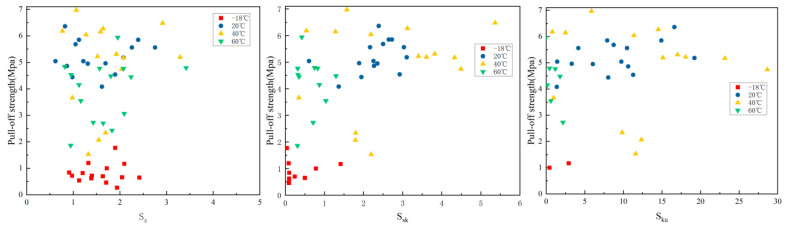
The relationship between three-dimensional maximum depth difference, skewness, peakedness, and drawing strength.

The relationship between S_z_, S_sk_, S_ku_, and pull-off strength at different curing temperatures is shown in Figure 7.

The Pearson correlation coefficients *r* between the S_z_ and POS (ranging from −18 °C to 60 °C) are −0.28, −0.18, 0.12, and −0.33, respectively, indicating no significant correlation at these temperatures. At high temperatures, an increase in S_z_ may slightly reduce strength, but this effect is not significant overall. The thermal contraction of the substrate at high temperatures may amplify the local defect effects of S_z_.

The Pearson correlation coefficients *r* (ranging from −18 °C to 60 °C) between the drawing strength of each temperature group and S_sk_ are −0.15, −0.12, −0.08, and −0.21, with no significant correlation observed. Although S_sk_ > 0 (indicating a peak structure predominates) theoretically enhances the mechanical interlocking effect, at low temperatures, the resin’s poor fluidity makes the overall surface roughness (e.g., S_a_/S_q_) the primary factor affecting strength, thus masking the independent effect of S_sk_. At moderate temperatures, the resin’s fluidity is optimized, and surface defects are filled, further reducing the impact of S_sk_. At high temperatures, although thermal contraction occurs, the symmetrical characteristics of S_sk_ do not significantly affect the distribution of thermal stress. S_sk_ reflects high distribution symmetry, while adhesion is more dependent on overall surface roughness (S_a_; S_q_) and effective contact area.

The Pearson correlation coefficients *r* (ranging from −18 °C to 60 °C) are −0.09, −0.14, −0.07, and−0.18, respectively, all showing no significant correlation. The S_ku_ is used to describe the sharpness of a highly distributed feature, whereas the strength of adhesion primarily depends on the effective contact area and the overall roughness index (S_a_; S_q_), which are not directly related to the sharpness of the distribution shape.

#### 3.2.4. The Relationship Between A_s_, A_r_, and POS at Different Curing Temperatures

**Figure 8:**
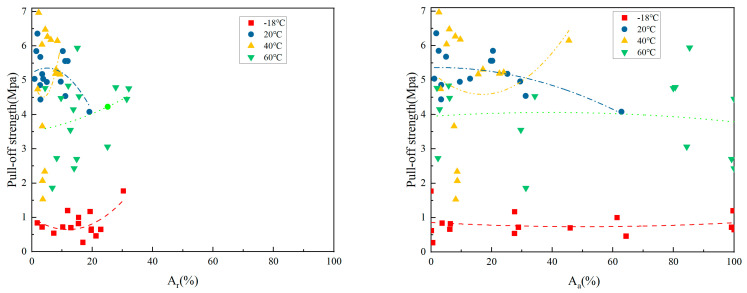
The relationship between relative and absolute proportion of hole area and drawing strength.

The relationship between the proportion of hole area and POS at different curing temperatures is shown in Figure 8.

According to the data analysis in Figure 8, the Pearson correlation coefficients for the relative proportions of drawing strength and pore area in each temperature group are *r* (−18 °C) ≈ −0.81, indicating a strong negative correlation; *r* (20 °C) ≈ −0.74, also showing a strong negative correlation; *r* (40 °C) ≈ −0.68, indicating a moderate negative correlation; and *r* (60 °C) ≈ −0.77, confirming a strong negative correlation.

At −18 °C, the increase in the proportion of concrete pores significantly reduces the concrete’s strength. During low-temperature curing, the poor fluidity of the resin reduces the effective contact area, leading to a sharp decline in adhesion. At 20 °C, the increase in pore proportion at room temperature leads to a decrease in strength. Although the resin’s fluidity improves at room temperature, the pores still hinder the mechanical interlocking between the coating and the substrate. At 40 °C, the impact of concrete pore proportion on strength is relatively reduced, and the resin’s filling ability is optimized, allowing some pores to be filled, thus mitigating the adverse effects of the high pore proportion. At 60 °C, a high pore proportion significantly reduces strength. During high-temperature curing, the resin’s contraction accelerates, making areas with a high pore proportion more prone to stress concentration, thereby increasing the risk of delamination.

The Pearson correlation coefficient *r* (−18 °C and 20 °C) between POS and the A_a_ shows a strong negative correlation; *r* (20 °C and 40 °C) is −0.91, *r* (40 °C and 60 °C) is −0.82, and *r* (60 °C and 80 °C) is −0.88. At low temperatures, the resin cannot fill the voids, leading to a high proportion of voids and nearly zero effective contact area. At room temperature, the resin partially fills the voids, but the high void area still forms localized weak points. At medium temperatures, the resin’s flowability is optimized, partially filling the voids, but the high void proportion still significantly reduces adhesion. High-temperature curing accelerates resin shrinkage, forming through defects in the high void areas, which severely weakens mechanical interlocking.

At all temperatures, the relative proportion of pores and their strength show a significant negative correlation; however, the absolute proportion of pores exhibits an extremely strong negative correlation. The pore ratio is a key parameter affecting adhesion, particularly at low and high temperatures, where it has the most significant impact on strength, necessitating strict control. In practical construction, the fluidity of the resin can effectively compensate for some pore defects, thus broadening the operational window for the process.

### 3.3. Model of Texture Feature Parameters and Epoxy Pull Strength at Different Temperatures

#### 3.3.1. Correlation Coefficient Between Three-Dimensional Parameters and Drawing Strength at Different Temperatures

**Figure 9:**
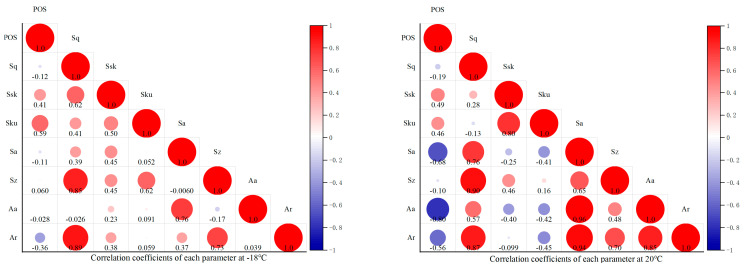

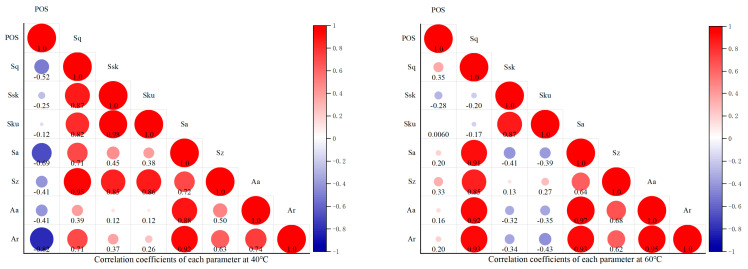
Correlation coefficient matrix of three-dimensional roughness parameters and drawing strength of epoxy coatings at different temperatures.

The correlation coefficient between three-dimensional roughness parameters and drawing strength at −18 °C, 20 °C, 40 °C, and 60 °C is shown in Figure 9. Univariate linear regression analysis results of various texture parameters and coating pull-off strength under different curing temperatures is shown in Table 2.

The graph shows that the tensile strength at low temperatures is most strongly correlated with S_ku_, while the other variables show no significant correlation. At room temperature, the significant variables are S_a_ and the absolute proportion of void area, while the insignificant variables include S_z_, S_sk_, and S_ku_. At medium to high temperatures, the significant variables are the relative proportion of voids and S_a_, while the insignificant variables include S_z_, S_sk_, and S_ku_. Given the strong correlations among parameters such as S_q_, S_a_, S_sk_, and S_ku_, parameters like S_a_, the absolute proportion of void area, and S_sk_ are used as variable parameters. In the high-temperature state, the significant variables are the absolute proportion of voids, the relative proportion of voids, S_a_, and S_q_; the insignificant variables include S_sk_, S_ku_, and S_z_. Due to the strong correlation between S_q_ and S_a_ (*r ≈* 0.915) and the absolute proportion of voids and the relative proportion of voids (*r ≈* 0.943), parameters like S_a_ and the absolute proportion of voids are selected as variable parameters. In addition, the significance of each parameter is analyzed through univariate linear regression, and significant parameters and moderately significant parameters are preferentially selected for the establishment of a nonlinear model.

#### 3.3.2. Pull-Off Strength of Coating at −18 °C

To quantitatively describe the relationship between the significant surface texture parameters and the pull-off strength (POS), multivariate nonlinear regression models [27,28,29,30,31] were developed using the experimental data. The model fitting was performed in Origin 2024 using the Curve Fitting Toolbox. The nonlinear least-squares method was employed to estimate the regression coefficients, ensuring the best fit by minimizing the sum of squared residuals.

This model quantitatively describes the relationship between significant variables in concrete surface roughness parameters and the adhesion performance (pull-off strength) of waterborne epoxy coatings. Formula (10) represents this multivariate nonlinear regression model, with its mathematical expression as follows:

(10) $$Y=\beta_{0}+\beta_{1}\ln\left(X_{1}\right)+\beta_{2}{\left(X_{2}\right)}^{1.5}+\beta_{3}\frac{1}{X_{1}}$$

where Y represents POS (MPa); X_1_ represents S_a_; and X_2_ represents A_a_. The regression coefficients for *β*_0_*~β*_3_ are 2.814; 0.572; −0.021; and −0.103, respectively. The model’s correlation coefficient *R*^2^ is 0.942, and Formula (11) is shown below.

(11) $$y=2.814+0.572\ln\left(S_{a}\right)-0.021{\left(A_{a}\right)}^{1.5}-\frac{0.103}{S_{a}}$$

The logarithmic and reciprocal formulas are used to simulate the nonlinear effects of S_a_. The logarithmic term indicates that the initial increase in S_a_ enhances drawing strength, but this effect gradually diminishes. The reciprocal term shows that when the roughness of S_a_ approaches zero, POS drops sharply. The 1.5th power term reveals a hyperlinear growth trend in the negative impact of pores as their proportion increases. Unlike the optimal value (S_a_ ≈ 0.375 mm) at room temperature, S_a_ needs to continuously increase in a low-temperature environment to compensate for the adverse effects of low-temperature brittleness. Pores are more sensitive to low temperatures, and for every 10% increase in pore ratio, the strength loss is about 30% higher at low temperatures compared to room temperature. In the room temperature model, S_a_ has a less significant impact on the pore area ratio, but S_a_ itself remains crucial.

#### 3.3.3. Pull-Off Strength of Coating at 20 °C

Following the same nonlinear regression methodology described in Section 3.3.2, the regression model (20 °C), Formula (12), is generated as follows:

(12) $$Y=\beta_{0}+\beta_{1}X_{1}+\beta_{2}{X_{1}}^{2}+\beta_{3}\sqrt{X_{2}}+\beta_{4}X_{1}X_{2}$$

where Y represents POS (MPa); X_1_ represents S_a_; and X_2_ indicates A_a_. The regression coefficients for *β*_0_ *to β*_4_ are 7.152; −18.642; 24.873; −0.418; and 0.327, respectively. The model’s correlation coefficient *R*^2^ is 0.96, with Formula (13) shown below.

(13) $$y=7.152-18.642S_{a}+24.873{S_{a}}^{2}-0.418\sqrt{A_{a}}+0.327S_{a}•A_{a}$$

The influence of S_a_ on drawing strength is nonlinear, as indicated by the coefficients of linear and quadratic terms. The optimal value obtained through differentiation is 0.375. To prevent strength reduction due to improper roughness, under the conditions of this study, we suggest maintaining S_a_ within a range of 0.35–0.40 mm. Since the strength decreases by approximately 0.8 MPa for every 10% increase in pore area, the pore ratio should be strictly controlled to no more than 15%.

#### 3.3.4. Pull-Off Strength of Coating at 40 °C

Following the same nonlinear regression methodology described in Section 3.3.2, the regression model (40 °C) Formula (14) is generated as follows:

(14) $$Y=\beta_{0}+\beta_{1}e^{{-X}_{1}}+\beta_{2}{\left(X_{2}\right)}^{0.5}+\beta_{3}{X_{3}}^{3}$$

where X_3_ represents S_sk_. The regression coefficients for *β*_0_*~β*_3_ are 8.327; 3.415; −1.074; and −0.029, respectively. The model’s correlation coefficient *R*^2^ is 0.981, with Formula (15) shown below.

(15) $$y=8.327+3.415e^{-S_{a}}-1.074\sqrt{A_{a}}-0.029{S_{sk}}^{3}$$

The exponential decay effect indicates that an increase in S_a_ leads to a rapid decrease in strength, consistent with the enhanced stress concentration at the interface under high temperatures. For every 10% increase in the pore ratio, the strength decreases by approximately 0.34 MPa (compared to 0.8 MPa at room temperature), indicating that the sensitivity to pores decreases in high-temperature environments. The cube of the S_sk_ represents how negative coefficients enhance the strength through negative S_sk_ (valley depth characteristics). However, when the negative S_sk_ is too high (S_sk_ < −1), adhesion drops sharply. In this environment, an increase in roughness continuously weakens the strength, and the symmetry of the surface morphology (S_sk_) has a significant impact.

#### 3.3.5. Pull-Off Strength of Coating at 60 °C

Following the same nonlinear regression methodology described in Section 3.3.2, the regression model (60 °C), Formula (16), is generated as follows:

(16) $$Y=\beta_{0}+\beta_{1}{X_{1}}^{0.5}+\beta_{2}{\left(X_{2}\right)}^{2}+\beta_{3}\frac{1}{\sqrt{X_{1}}}$$

The regression coefficients for *β*_0_*~β*_3_ are 6.214; 2.835; −0.008; and −1.047, respectively. The model’s correlation coefficient *R*^2^ is 0.953, indicating an extremely high fit with the formula shown below.

(17) $$y=6.214+2.835\sqrt{S_{a}}-0.008{A_{a}}^{2}-\frac{1.047}{\sqrt{S_{a}}}$$

The square root term indicates that S_a_ has a bidirectional effect, meaning that a moderate increase in S_a_ can enhance the drawing strength. The reciprocal of the square root term shows that when S_a_ is too low and approaches zero, the strength drops sharply. The quadratic term reflects that the negative impact of pore defects accelerates as their proportion increases. At 60 °C, it is necessary to balance the improvement in roughness with the risk of stress concentration. At this temperature, the negative impact of pores accelerates more significantly; for every 10% increase in the pore ratio, the strength loss is about 45% higher than at room temperature. As the temperature rises from 20 °C to 60 °C, the positive effect of S_a_ gradually diminishes, and the optimal S_a_ value shifts towards a lower range. It is recommended that the S_a_ range be between 0.20 and 0.35 at high temperatures, slightly lower than the approximately 0.35 at room temperature.

## 4. Conclusions

This paper analyzes the surface morphology characteristics of concrete substrates and its influence on the adhesion performance of an epoxy coating at different temperatures; reveals the internal relationship between surface treatment process, morphology parameters, and drawing strength; and establishes a multivariate mathematical model. The main conclusions are as follows:

(1) Grinding treatments significantly altered the microstructure of concrete surfaces. While grinding introduced scratches and exposed more penetration channels, enhancing the mechanical interlocking effect of the coating, the roughness index of silica fume (indicating large pore defects) did not change significantly, indicating that the surface treatments primarily affected the microstructure rather than the macrostructure. Three-dimensional modeling further confirmed that the actual effects of pore expansion and scratch increase after grinding were consistent with the negative correlation of three-dimensional morphology parameters (S_a_, S_q_).
(2) Temperature regulation of the morphology–strength relationship: At low temperatures (−18 °C), the resin’s poor fluidity prevented it from filling the peaks and valleys on rough surfaces, leading to a more severe negative impact on S_a_ and the proportion of pores. It is recommended to keep S_a_ < 0.35 and the absolute pore ratio < 15%. At room temperature (20 °C), the resin’s fluidity improved, but high roughness (S_a_ > 0.45) still limited penetration. This model’s optimal S_a_ value of 0.375 results in a tensile strength exceeding 5.5 MPa. At medium to high temperatures (40–60 °C), the resin’s filling ability was optimized, partially compensating for rough defects. However, high temperatures accelerated curing shrinkage, and the negative effects of S_a_ and the pore ratio reappeared. It is necessary to balance roughness with thermal stress risks.
(3) Based on the nonlinear regression analysis, a quantitative model has been established for the relationship between drawing strength and morphological parameters at different temperatures. In the 20 °C model, strength is quadratic with S_a_, with the optimal S_a_ value being approximately 0.375; for every 10% increase in the porosity ratio, the strength decreases by 0.8 MPa. In the −18 °C model, strength increases logarithmically with S_a_, but the sensitivity to pore defects is higher, so controlling the porosity ratio should be prioritized. In the 60 °C model, the combined effect of the S_a_ index and the square root of pore size suggests that S_a_ should be kept below 0.35 to avoid thermal stress concentration.
(4) In actual concrete coating applications, it is recommended to optimize the surface roughness through grinding, strictly control the absolute proportion of holes, and prioritize using preheated or low-viscosity resin in a low-temperature environment to improve permeability.

This study’s findings are specific to the coating formulation and surface treatment methods employed. Future work should extend this approach to other coating systems and surface preparation techniques. Furthermore, advanced characterization techniques such as micro-computed tomography could be integrated to obtain more detailed three-dimensional pore network information and validate the pore area parameters defined in this study.

## Figures and Tables

**Table 1:** SRI (mm) of different test areas.

Sequence Number	Surface 1 Initial SRI	Surface 1 Grind SRI	Surface 2 Initial SRI	Surface 2 Grind SRI	Surface 3 Initial SRI	Surface 3 Grind SRI	Surface 4 Initial SRI	Surface 4 Grind SRI
1	0.4610	0.4870	0.1908	0.2091	0.0804	0.0378	0.2557	0.2340
2	0.1012	0.0806	0.3534	0.3389	0.2163	0.0522	0.2610	0.2275
3	0.0567	0.0871	0.4666	0.4879	0.1531	0.1216	0.3697	0.2336
4	0.1101	0.1223	0.3818	0.3649	0.0994	0.1240	0.1421	0.2151
5	0.4127	0.3620	0.2536	0.2622	0.2964	0.2412	0.2095	0.1697
6	0.0667	0.0458	0.2434	0.2110	0.0506	0.0746	0.2604	0.2637
7	0.0602	0.0239	0.3258	0.3139	0.0404	0.0558	0.2430	0.1943
8	0.1295	0.0564	0.3057	0.2943	0.0466	0.0542	0.2091	0.2770
9	0.2865	0.2440	0.2660	0.2419	0.1389	0.1296	0.1208	0.1927
10	0.3918	0.1926	0.1971	0.1716	0.1593	0.1101	0.1816	0.195
11	0.1756	0.1612	0.3620	0.3925	0.0817	0.0627	0.3512	0.4114
12	0.2175	0.1835	0.2497	0.2825	0.0737	0.0981	0.3962	0.3989
13	0.1309	0.1271	0.2435	0.2756	0.1334	0.0513	0.1975	0.2322
14	0.2269	0.0958	0.2682	0.1940	0.1385	0.1810	0.2314	0.2277
15	0.1745	0.1662	0.1962	0.1737	0.1534	0.1544	0.1834	0.1566

**Table 2:** Summary of univariate linear regression analysis results of various texture parameters and coating pull-off strength under different curing temperatures (significance level α = 0.05).

Parameter	Correlation Coefficient (*p*-Value), n = 15			
	−18 °C	20 °C	40 °C	60 °C
S_a_	−0.113 (0.772)	−0.678 (0.044)	−0.690 (0.040)	0.195 (0.614)
S_q_	−0.118 (0.764)	−0.194 (0.618)	−0.516 (0.153)	0.353 (0.356)
S_sk_	0.414 (0.267)	0.489 (0.183)	−0.247 (0.520)	−0.281 (0.468)
S_ku_	0.585 (0.098)	0.458 (0.215)	−0.124 (0.750)	0.006 (0.987)
S_z_	0.060 (0.867)	−0.103 (0.792)	−0.412 (0.270)	0.325 (0.392)
A_a_	−0.028 (0.943)	−0.795 (0.011)	−0.412 (0.270)	0.158 (0.678)
A_r_	−0.361 (0.339)	−0.564 (0.115)	−0.818 (0.007)	0.199 (0.607)

## Data Availability

The original contributions presented in this study are included in the article. Further inquiries can be directed to the corresponding author.
